# Isolation and Characterization of a Novel *Autographiviridae* Phage and Its Combined Effect with Tigecycline in Controlling Multidrug-Resistant *Acinetobacter baumannii*-Associated Skin and Soft Tissue Infections

**DOI:** 10.3390/v14020194

**Published:** 2022-01-20

**Authors:** Phitchayapak Wintachai, Komwit Surachat, Kamonnut Singkhamanan

**Affiliations:** 1 School of Science, Walailak University, Nakhon Si Thammarat 80161, Thailand; 2 Division of Computational Science, Faculty of Science, Prince of Songkla University, Hat Yai 90110, Thailand; komwit.s@psu.ac.th; 3 Molecular Evolution and Computational Biology Research Unit, Faculty of Science, Prince of Songkla University, Hat Yai 90110, Thailand; 4 Department of Biomedical Sciences and Biomedical Engineering, Faculty of Medicine, Prince of Songkla University, Hat Yai 90110, Thailand; skamonnu@medicine.psu.ac.th

**Keywords:** *Autographiviridae* phage, *Acinetobacter baumannii*, bacteriophage, antibacterial activity, biofilms, synergistic activity, phage therapy

## Abstract

Multidrug-resistant *Acinetobacter baumannii* (MDR *A. baumannii*) is one of the ESKAPE pathogens that restricts available treatment options. MDR *A. baumannii* is responsible for a dramatic increase in case numbers of a wide variety of infections, including skin and soft tissue infections (SSTIs), resulting in pyoderma, surgical debridement, and necrotizing fasciitis. To investigate an alternative medical treatment for SSTIs, a broad range lytic *Acinetobacter* phage, vB _AbP_ABWU2101 (phage vABWU2101), for lysing MDR *A. baumannii* in associated SSTIs was isolated and the biological aspects of this phage were investigated. Morphological characterization and genomic analysis revealed that phage vABWU2101 was a new species in the *Friunavirus*, *Beijerinckvirinae*, family *Autographiviridae*, and order *Caudovirales*. Antibiofilm activity of phage vABWU2101 demonstrated good activity against both preformed biofilms and biofilm formation. The combination of phage vABWU2101 and tigecycline showed synergistic antimicrobial activities against planktonic and biofilm cells. Scanning electron microscopy confirmed that the antibacterial efficacy of the combination of phage vABWU2101 and tigecycline was more effective than the phage or antibiotic alone. Hence, our findings could potentially be used to develop a therapeutic option for the treatment of SSTIs caused by MDR *A. baumannii.*

## 1. Introduction

*Acinetobacter baumannii* (*A. baumannii*), an opportunistic Gram-negative bacterium, has emerged as a superbug that is a significant multidrug-resistant (MDR) nosocomial pathogen. The increasing capacity of *A. baumannii* to develop resistance to multiple antimicrobial agents has been reported continuously, causing significant health problems, an increase in morbidity and mortality, and treatment failures [1]. MDR *A. baumannii* causes many types of infections, such as respiratory infections, bloodstream infections, and urinary tract infections. Recent reports have suggested that MDR *A. baumannii* is also associated with skin and soft tissue infections (SSTIs) [2,3], and *A. baumannii* infections have been identified as a serious problem in diabetic foot ulcers [4,5]. Even though *A. baumannii* is an opportunistic pathogen primarily associated with hospital-acquired infections, *A. baumannii*-associated SSTIs in healthy patients have been reported [6]. The induction of MDR *A. baumannii* infections in SSTIs causes chronic infections, which often contribute to delayed wound healing, pyoderma, necrotizing soft tissue, skin-graft failure, necrotizing fasciitis, and sepsis [7,8].

MDR *A. baumannii* can adhere and persist on abiotic and biotic surfaces as biofilms, which contributes to the increase in bacterial survival, spread and long-term persistence [9]. Moreover, biofilms of MDR *A. baumannii* are associated with the tolerance to germ-killing environments and disinfectants, resulting in the difficulty of their management and the enhancement of antibiotic-resistant bacteria. Previous reports have shown that the adherence of *A. baumannii* to epithelial cells also causes biofilm formation, increasing persistent bacterial infections and skin infections or skin diseases [10,11].

Tigecycline, the first drug in the glycylcycline class of antibiotics, is the approved antibiotic for treating SSTIs. Tigecycline is widely used for the treatment of urinary tract infections, prostatitis [12], septicemia [13], intra-abdominal infections [14], inflammatory diseases [15], and SSTIs [16]. In addition, tigecycline is often used to treat more severe aerobic Gram-negative bacilli infections, including *A. baumannii* infections [17,18]. Because tigecycline is a time-dependent agent with concentration-dependent killing activity, a higher initial concentration and multiple dose regimens of antibiotics are required to treat patients [19]. The overuse of antibiotics, including tigecycline, is associated with an increased risk of adverse effects [20]. The development of adverse drug reactions is well documented. Nausea, vomiting, dizziness, sleep problems, and pain/swelling at the injection site can develop in a short time period after tigecycline administration. Some examples of mild adverse effects associated with the use of tigecycline are a higher risk for hepatitis, pancreatitis, vertigo, hearing loss, lupus erythematous, and hyperpigmentation [21]. Currently, the outbreaks of *A. baumannii* resistant to tigecycline are becoming increasingly prevalent [22,23]. The development of new treatments or medications should be considered. Several alternatives to conventional antibiotics for SSTIs have been discovered, including the use of phages to treat bacterial infections.

Bacteriophages or phages are the viruses of bacteria that specifically infect their hosts. In the pre-antibiotic era, phages were commonly used to treat bacterial infections in people and animals [24]. Phages were replaced by antibiotics when antibiotics were discovered. Because antibiotic resistance has become more concerning globally, the rise of this problem has resulted in a marked increase in interest in phage therapy [25]. The isolation and characterization of phages specific to MDR *A. baumannii* have been reported continuously [26]. Two novel phages infecting MDR *A. baumannii* were characterized and evaluated in mice [27]. A phage cocktail composed of *A. baumannii* phages was proven effective as a biofilm control agent in a human urine model, and synergistic effects of the cocktail and antibiotics were evaluated [28]. Interestingly, a clinical trial of phage therapy against MDR *A. baumannii* craniectomy site infection has been reported [29]. A phage cocktail was successfully used to treat a patient with a disseminated resistant MDR *A. baumannii* infection [30]. Nevertheless, few studies on *Acinetobacter* phage-related SSTIs have been reported. A *Siphoviridae* phage was used to successfully control MDR *A. baumannii*-associated wound infections in uncontrolled diabetic rats [31]. A phage cocktail of AB-Navy1, AB-Navy2, AB-Navy3, and AB-Navy4 was effective against *A. baumannii* wound infections in mice [32]. These phenomena highlight the possibility of using phages to treat *A. baumannii*-associated SSTIs. In Thailand, a few studies on phage isolations have been reported, and there are no commercial phage products available nowadays [33,34,35]. Due to the emergence of MDR bacteria in Thailand similar to other countries worldwide, alternative strategies to control MDR bacterial infections, including phage and the combination of phages with other antibiotics, are attractive to investigate among researchers. Consequently, phage studies might shed more light on their potential application in therapy to combat MDR bacteria.

In this study, we focused on the isolation and characterization of a novel *Autographiviridae* phage that infected MDR *A. baumannii* associated with SSTIs. Host range, biological aspects of phage, complete genome sequencing, antibiofilm efficacy of phage, and synergistic activity of phage and tigecycline against planktonic and biofilm cells were evaluated.

## 2. Materials and Methods

### 2.1. Culture Media and Antibiotic

Tryptic Soy Agar (TSA), Typtic Soy Broth (TSB), Mueller Hinton Agar (MHA), and Mueller Hinton Broth (MHB) were obtained from Becton, Dickinson and Company (Franklin Lakes, NJ, USA). Tigecycline was purchased from Pfizer Inc. (Philadelphia, PA, USA).

### 2.2. Bacterial Strains and Growth Conditions

Twenty clinical isolates from MDR *A. baumannii*-associated SSTIs were kindly provided by the Molecular Microbiology laboratory, Faculty of Medicine, Prince of Songkla University, Thailand, and the bacterial strains were isolated from the specimens of routine laboratory services, Songklanagarind Hospital, Songkhla Province, Thailand. *Klebsiella pneumoniae (K. pneumoniae)* and methicillin-resistant *Staphylococcus aureus* (MRSA) clinical isolates were obtained from the same source. All bacterial isolates were cultured on TSA and TSB followed by incubation at 37 °C.

### 2.3. Phage Isolation, Purification, and Phage Morphology by Transmission Electron Microscopy (TEM)

Phage was enriched from environmental water samples, which were collected from the San Saep canal, Bangkok, Thailand. Samples were centrifuged to remove the debris at 6400× *g* for 15 min at 4 °C. The supernatant was filtered through a syringe sterile filter 0.22 μm (GVS, CA, USA). Ten milliliters of supernatant were mixed with 10 mL of TSB and 200 µL of MDR *A. baumannii* ABPW0185, a host strain. The mixture was incubated overnight at 37 °C with shaking at 150 rpm and then centrifuged at 6400× *g* for 15 min at 4 °C. The supernatant was collected and filtered through a sterile 0.22 μm filter. The phage was detected by a double agar overlay plaque assay. The phage was purified, amplified, and concentrated as described previously [28,34]. A single plaque was extracted from the soft top agar by a sterile tip. The plaque was soaked in 500 μL sterile SM buffer (0.1 M NaCl, 8 mM MgSO_4_, 7H_2_O, 50 mM Tris-HCl pH 7.5) overnight at 4 °C. The supernatant was serially diluted 10-fold in SM buffer and 200 μL of diluted phage was supplemented with 200 μL of log phase of MDR *A. baumannii.* After incubation for 15 min, the mixture was mixed with top agar and poured onto a TSA plate. Plaques were observed on the plates after incubation overnight at 37 °C. These steps were repeated five times for phage purification. To amplify the phage, the phage was amplified by a double agar overlay plaque assay. The phage was eluted from the semi-confluent plates by adding SM buffer followed by incubation at 4 °C overnight. The supernatant was collected and then centrifuged at 6400× *g* for 20 min at 4 °C. After filtration through a 0.22 μm pore filter, the supernatant was collected and then concentrated by Amicon^®^ Ultra-15 centrifugal filter units with Ultracel 100 kDa membrane (Millipore, MA, USA) [36]. The concentrated phage lysate was collected as phage stock and the number of phage particles in the stock was determined by phage titration as described elsewhere [37,38]. The phage stock was kept at 4 °C until used. TEM was performed to observe the phage morphology using a transmission electron microscope as described previously [34]. The obtained phage was dropped onto a copper grid surface and negatively stained with 2% (vol/vol) uranyl acetate (pH 6.7). The micrographs of phage were taken under a JEOL JEM-2010 transmission electron microscope at an acceleration voltage of 160 kV.

### 2.4. Host Range Analysis and Efficiency of Plating (EOP)

The ability of the obtained phage to infect different bacterial strains was tested against 20 clinical isolates of MDR *A. baumannii*-associated SSTIs. The host range was also evaluated in *K. pneumoniae* and MRSA using the standard spot test. In brief, 200 µL of each log phase bacterial isolate was mixed with the melt top agar and poured onto a TSA plate. The phage was serially diluted in SM buffer and 10 µL of the diluted phage (10^4^ PFU/mL) was dropped onto the overlaid top agar containing the tested bacterial strain. After incubation overnight at 37 °C, the presence of a clear zone was observed on the bacterial lawn. The bacterial strains that could be lysed by phage vABWU2101 were selected for EOP analysis. In brief, a serial dilution of phage was prepared in SM buffer and then mixed with the different bacterial strains followed by a double agar overlay plaque assay. After incubation overnight at 37 °C, the plaques were counted. The ratio of the number of plaques produced on the bacterial lawn of the test bacterial strain to the number of plaques produced on the lawn of the host bacterial strain was reported as an EOP value. The EOP values were classified into 4 groups: high production (a ratio ≥ 0.5), moderate production (a ratio 0.1 ≤ EOP < 0.5), low production (a ratio 0.001 < EOP < 0.1), and no production (a ratio ≤ 0.001). The experiments were undertaken independently in duplicate with duplicate plaque assay.

### 2.5. Phage Adsorption Rate to Bacterial Host Cells

The adsorption assay was carried out to determine the kinetics of phage adsorption, and the adsorption rate constant *k*, in mL/min, was calculated as described previously [34,39]. In brief, MDR *A. baumannii* was diluted and then infected with the phage at an MOI of 1. At 0–10 min, samples were collected every minute and the supernatant was collected every 5 min at 10–30 min. The samples were added into the prechilled TSB supplemented with chloroform. The unabsorbed phage particles were enumerated by a double agar overlay plaque assay. The experiments were undertaken independently in duplicate with duplicate plaque assay.

### 2.6. One Step Growth Assay

A one-step growth experiment was undertaken as described previously [34]. In brief, MDR *A. baumannii* was cultured to the log phase of the growth curve and then adjusted to a final OD600 of 0.1. Bacterial culture was centrifuged at 6000× *g* for 10 min at 4 °C and the pellet was resuspended in TSB. After being supplemented with phage vABWU2101 at an MOI of 1, the mixture was incubated for 15 min and then centrifuged to remove the unabsorbed phage at 6000× *g* for 20 min at 4 °C. The bacterial pellet was resuspended in TSB followed by incubation at 37 °C. Samples were taken at 10 min intervals until 120 min. Supernatants were filtered, diluted, and then titrated by the soft-agar overlay method. Burst size was calculated as the ratio of phage titer to the number of initial infected bacterial cells. The experiments were undertaken independently in duplicate with duplicate plaque assay.

### 2.7. Lytic Profile of Bacteria after Phage Infection

The bacteriolytic efficacy of phage vABWU2101 was evaluated as described previously [34]. MDR *A. baumannii* was cultured overnight and the optical density at 600 nm was adjusted to 0.1. MDR *A. baumannii* was infected with phage vABWU2101 at MOIs of 0.01, 0.1, 1, and 10, followed by incubation at 37 °C with agitation. As a control sample, MDR *A. baumannii* was incubated with TSB without the phage. Bacterial growth was monitored by measuring at OD600nm every hour for 10 h. The experiments were undertaken independently in duplicate with duplicate assay.

### 2.8. The Influence of Temperature, pH, and UV Radiation on Phage Viability

The stability of phage vABWU2101 under different temperatures (−80, −20, 4, 25, 37, 50, 60, 70, 80, and 90 °C) and pH values (1–14) was tested according to a previous report [34]. In brief, the phage suspension was diluted to a final concentration of 10^8^ PFU/mL. The phage was incubated at a specific temperature and pH for 2 h. The phage in the thermal test was placed in an ice-water bath, and the phage at different pH values was neutralized to pH 7. The viability of the phage was determined by a double agar overlay plaque assay. The phage, incubated at 25 °C or pH 7, was used as a control for the thermal and pH tests, respectively. For the thermal stability test, the phage incubated at 4 °C to 90 °C was diluted in SM buffer, and the phage tested at −80 and −20 °C was diluted in SM buffer supplemented with glycerol to a final concentration of 50% glycerol [40]. The influence of UV radiation on phage vABWU2101 stability was also determined. In brief, the phage suspension was diluted to a final concentration of 10^8^ PFU/mL and then added to the open Petri dishes. The dishes were incubated on ice and placed 30 cm away from the UV-C light source. After exposure to the UV-C light, the phage supernatant was collected every 10 min for 1 h followed by titration. The experiments were undertaken independently in duplicate with duplicate plaque assay.

### 2.9. Long-Term Stability

The stability of the phage vABWU2101 during long-term storage was determined by a double agar overlay plaque assay. The phage was diluted to a final concentration of 10^8^ PFU/mL and kept at 4 °C. The supernatant was collected for determination of titer every month for 6 months. The experiments were undertaken independently in duplicate with duplicate plaque assay.

### 2.10. Whole-Genome Characterization and Analysis

Whole genome sequencing was conducted commercially on the Illumina sequencing platform (Novogene, Beijing, China). Genomic DNA was extracted and randomly fragmented by sonication. The DNA fragments were end polished, A-tailed, and ligated to the full-length adapters of Illumina sequencing. After PCR amplification with P5 and indexed P7 oligos, the libraries were purified with AMPure XP system, and the size distribution of the libraries was checked by the Agilent Bioanalyzer (Agilent Technologies, CA, USA). The libraries were quantified by real-time PCR followed by sequencing. For the analysis, raw data were filtered to remove the low-quality read data. The assembly procedure and fill gap process were performed to generate the genomic sequences, and the assembly results were evaluated. Filtered reads were assembled using SPAdes de novo assembly (version 3.11.1) [41]. The locations of genes that coded for proteins, tRNA genes, and rRNA genes were predicted by Prokka (v1.12) [42]. The functions were then annotated by BLAST and RAST version 2.0 server [43,44]. The DNA packaging mechanism of phage vABWU2101 was evaluated by the PhageTerm analysis [45]. The genome maps were plotted using Clinker [46].

The whole-genome phylogeny was constructed by the Geneious server [47]. In brief, the complete genome sequences were aligned using the NCBI Multiple Sequence Alignment Viewer and then constructed the neighbor-joining phylogenetic tree based on the Jones–Taylor–Thornton (JTT) model with 500 bootstrap replications [48,49]. The tRNA genes in genomic sequences were also searched using the tRNAscan-SE web server [50].

### 2.11. Phylogenetic Tree Analysis of Specific Genes

RNA polymerase from BlastX search and phage terminase large subunit proteins from RAST annotation were selected to construct the phylogenetic trees by the Geneious server. The sequences were aligned using MUSCLE and then constructed the neighbor-joining phylogenetic tree based on the JTT model with 1000 bootstrap replications.

### 2.12. Anti-Biofilm Activity of Phage

The activity of phage vABWU2101 in preventing biofilm formation and removing biofilms was evaluated by crystal violet staining and determination of cell viability as described previously [34,51]. To assess the efficacy of phage vABWU2101 in preventing biofilm formation, MDR *A. baumannii* was cultured at 37 °C overnight and then diluted to 10^8^ CFU/mL. One hundred microliters of MDR *A. baumannii* were added to a flat-bottomed 96-well microtiter plate and then supplemented with the indicated number of phage vABWU2101 (10^1^ to 10^8^ PFU/well). The plates were incubated for 2 days at 37 °C without agitation. For assessing the biofilm removal efficiency, MDR *A. baumannii* was cultured at 37 °C overnight and then diluted to 10^8^ CFU/mL. MDR *A. baumannii* was added to a flat-bottomed 96-well microtiter plate and then incubated at 37 °C without agitation. On Day 2 post incubation, the bacterial suspension was removed, and the wells were washed twice with PBS. The indicated number of phage vABWU2101 (10^1^ to 10^8^ PFU/well) was added to the wells, and the plates were incubated for 24 h at 37 °C without agitation. To evaluate the biofilm biomass by crystal violet staining, the supernatant in the wells was removed and then washed twice with PBS. The wells were air-dried and stained with 200 μL of 0.1% crystal violet (Sigma-Aldrich Chemicals, St. Louis, MO, USA). Due to the light sensitivity of crystal violet, the plates were incubated at room temperature in the dark for 30 min. The crystal violet was removed, and the wells were washed with PBS four times. The wells were air dried, and bound crystal violet in the wells was solubilized using 200 μL of 95% ethanol. The absorbance of the crystal violet was measured at 600 nm as biofilm biomass. The viable cells in the biofilm were also evaluated with the colony counting method. The supernatant in the wells was discarded, and the wells were then washed twice with PBS. One hundred microliters of PBS were added to the wells to resuspend the biofilm. The supernatant was serially diluted in PBS and dropped to the plates. The plates were incubated overnight at 37 °C and the colonies were counted as the number of biofilm viable cells. The experiments were undertaken independently in triplicate with duplicate assay.

### 2.13. Determination of the Antibacterial Activity of Tigecycline against MDR A. baumannii

The activity of tigecycline against MDR *A. baumannii* was assessed by determination of minimal inhibitory concentration (MIC) and minimal bactericidal concentration (MBC). The MIC assay was tested by a modified broth microdilution method that followed the Clinical and Laboratory Standard Institute (CLSI) guidelines, followed by evaluation of MBC [52,53]. In brief, an exponential-phase MDR *A. baumannii* culture was adjusted for a final OD600 of 0.1, corresponding to approximately 1 × 10^8^ CFU/mL, and the bacterial suspension was diluted to 1 × 10^6^ CFU/mL. Tigecycline was added to 96-well plates, and then a twofold serial dilution of tigecycline in MHB was prepared. A total of 100 µL of bacterial suspension was added to the wells, followed by supplementation with 50 µL of MHB. After incubation at 37 °C for 18 h, the MIC value was determined by resazurin staining. Ten microliters of resazurin (Sigma-Aldrich Chemicals, St. Louis, MO, USA) was added to the wells and then incubated in the dark for another 2 h. The color changes were observed and recorded. The lowest concentration prior to the color change was considered the MIC value. For determination of MBC, ten microliters of the concentrations greater than or equal to the MIC value were dropped onto MHA plates, followed by incubation at 37 °C overnight. Colony growth was observed, and the lowest concentration at which there was no bacterial growth was considered the MBC value. Experiments were undertaken independently in triplicate with duplicate assay.

### 2.14. Evaluation of the Combined Effects of Phage vABWU2101 and Tigecycline against MDR A. baumannii

The synergistic antibacterial activity of phage vABWU2101 and tigecycline against MDR *A. baumannii* was determined. In brief, MDR *A. baumannii* was cultured at 37 °C until the log phase of bacterial culture was reached. The bacterial suspension was adjusted to an OD600 of 0.1 and then diluted to a final concentration of approximately 1 × 10^6^ CFU/mL. In 96-well microtiter plates, tigecycline was serially diluted in MHB, and then 50 µL of phage was added at an MOI of 0.1 or 1 diluted in MHB. One hundred microliters of MDR *A. baumannii* was added to the wells. The antibacterial activities of phage vABWU2101 alone (MOI of 0.1 or 1), and tigecycline alone were assessed in parallel. After incubation at 37 °C for 18 h, MIC and MBC tests were evaluated using the protocol described above. Experiments were undertaken independently in triplicate with duplicate assay. The antibacterial combinations were assessed based on the fractional inhibitory concentration (FIC) index. For the FIC index calculation, the FIC index value was calculated by the following equation: ∑FIC = FIC of antibiotic + FIC of phage = MIC of tigecycline in combination/MIC of tigecycline alone + MIC of phage in combination/MIC of phage alone. The results were interpreted according to FIC indexes as follows: synergistic (∑FIC: ≤0.5), additive (∑FIC: >0.5 and ≤1), indifferent (∑FIC: >1 and ≤4), and antagonistic (∑FIC: >4) [54].

### 2.15. Killing Kinetics of the Antibacterial Activity of Phage vABWU2101 and Tigecycline Combination

The combined effects of phage vABWU2101 and tigecycline on the bacterial growth curve were assessed in 96-well plates. In brief, MDR *A. baumannii* was cultured in MHB at 37 °C overnight and then diluted. The measurement of OD600 was used to adjust the bacterial suspension. Tigecycline was serially diluted twofold, and 50 µL of tigecycline at 1/2× to 1/32× MIC were then added to 96-well microtiter plates. Phage vABWU2101 was diluted in MHB, and 50 µL of the phage at an MOI of 1 was added to the wells. Subsequently, the suspension was supplemented with 100 µL of diluted MDR *A. baumannii* followed by incubation at 37 °C. The killing kinetics were observed by measurement of the OD600 every hour for 12 h. The plates were incubated at 37 °C overnight. At 24 h post treatment, bacterial growth was measured by the OD600, and cell viability was observed by colony counting. MDR *A. baumannii* was diluted in TSB, and 10 µL of bacterial suspension was added to the MHA plates. After incubation at 37 °C overnight, the colonies were counted. Experiments were undertaken independently in triplicate with duplicate assay.

### 2.16. Activity of the Phage and Tigecycline Combination against Biofilms

The activity of the combination of phage vABWU2101 and tigecycline in preventing biofilm formation and removing biofilms was evaluated by crystal violet staining and determination of cell viability as described above with some modifications [34,51]. For efficacy in preventing biofilm formation, MDR *A. baumannii* was added to flat-bottomed 96-well microtiter plates and then supplemented with phage vABWU2101 at an MOI of 1 and tigecycline at 1/32–4× MIC. The plates were incubated for 2 days at 37 °C without agitation. For biofilm removal efficiency, MDR *A. baumannii* was added to the 96-well microtiter plates and then incubated at 37 °C without agitation for 2 days. The culture supernatant was discarded, and the wells were then washed twice with PBS. The phage alone, tigecycline alone, or combinations of phage vABWU2101 and tigecycline at 1/32–4× MIC were added to the wells, followed by incubation for 24 h at 37 °C without agitation. MDR *A. baumannii* without treatment was used as a control. Measurements of biofilm biomass were carried out with crystal violet staining, and the viable biofilm cells were evaluated by the colony-counting method. The experiments were undertaken independently in triplicate with duplicate assay.

### 2.17. Scanning Electron Microscopy (SEM)

The morphology of MDR *A. baumannii* after treatment with phage vABWU2101, tigecycline, and the combination of phage and tigecycline was visualized under SEM. In brief, MDR *A. baumannii* was cultured at 37 °C until the log phase was reached. MDR *A. baumannii* was incubated with only phage vABWU2101 (an MOI of 1), only tigecycline (1/32× MIC), and a combination of phage and tigecycline, individually at 37 °C for 2 h. At the indicated time point, the cells were collected by centrifugation at 12,000× *g* for 5 min. The supernatant was discarded, and the cells were then washed twice with PBS. Subsequently, the cells were fixed with 2.5% glutaraldehyde in 0.1 M PBS. The cells were washed with 0.1 M sodium phosphate buffer and then incubated in 1% OsO_4_ in DI water. After washing with DI water, the cells were dehydrated in a series of ethanol solutions (20, 40, 60, 80, and 100%). The cells were dried by a critical point dyer technique and then coated with gold. The cell morphology was observed under a field emission scanning electron microscope (Merlin compact, Zeiss, EDX (Oxford, Aztec), EBSD (Oxford, Nordlys Max)).

### 2.18. Statistical Analyses

Graphs and statistical analysis of significance were analyzed using the GraphPad Prism program (GrapPad Software, San Diego, CA, USA). An unpaired *t*-test was undertaken for statistical analysis and a *p*-value of <0.05 was considered to be statistically significant.

### 2.19. Nucleotide Sequence Accession Number

The complete genome sequences of phage vABWU2101 were deposited in the NCBI database (GenBank accession number: OK546191.1).

## 3. Results

### 3.1. Phage Isolation, Purification and Physical Characterization

**Figure 1:**
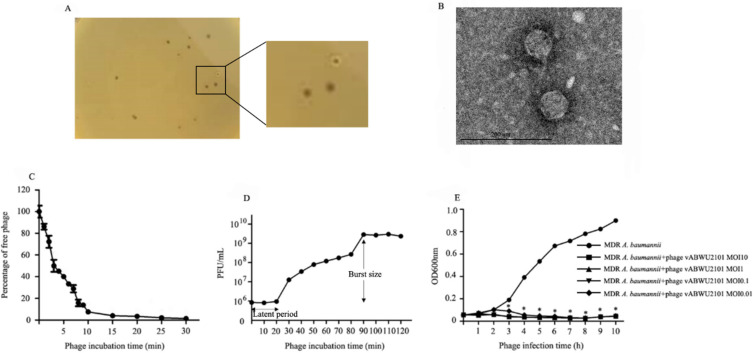
Isolation, morphology, and biological properties of phage vABWU2101. (**A**) Plaque morphology of phage vABWU2101; (**B**) transmission electron micrograph of vABWU2101. Scale bar indicates 200 nm. (**C**) Adsorption rate of phage vABWU2101 to host bacterial strain. (**D**) One-step growth curve of phage vABWU2101 to host bacterial strain. The latent period and burst size of phage vABWU2101 were estimated. (**E**) In vitro planktonic cell lysis assay of phage vABWU2101 at different MOIs against host bacterial strain. Experiments were undertaken independently in duplicate with duplicate assay. The data show the mean ± SD (* *p* value < 0.05).

A phage was enriched and isolated from environmental water. The phage produced small clear plaques 1–2 mm in diameter surrounded by a halo in a lawn of MDR *A. baumannii* (Figure 1A). The morphology of the phage particles was characterized under TEM. The phage had a hexagonal head diameter of 56.73 ± 1.92 nm from vertex to vertex and a short noncontractile tail (Figure 1B). Morphological characterization identified that the phage belonged to the family *Autographiridae* or *Podoviridae*, which are composed of short-tail phages. The phage was named based on morphology following the Kropinski system [55], and the phage was designated *Acinetobacter* phage vB _AbP_ABWU2101 and called phage vABWU2101 for short.

### 3.2. Host Range Activity Determination and EOP Assay

The host range activity of phage vABWU2101 was determined by a spot test and EOP analyses. The spot test assay showed that phage vABWU2101 had the ability to produce a lytic zone against 70% of the tested MDR A. baumannii-associated SSTIs isolates. There was no lytic activity of phage vABWU2101 against K. pneumoniae and MRSA (Table 1). The MDR *A. baumannii* isolates that were susceptible to phage vABWU2101 infection were further assessed by EOP analysis. The results showed high, medium, and low productive infection on MDR *A. baumannii* associated SSTIs in 6, 5, and 3 MDR *A. baumannii* isolates, respectively.

### 3.3. Phage Characterization

The adsorption velocity of phage vABWU21001 was evaluated by a phage adsorption assay. More than 60% of the phage was adsorbed to the host cell within 5 min, and the percentage of adsorption was nearly 100% at 15 min post-incubation (*p* = 0.0033). The adsorption rate constant *k* was 2.59 × 10^−8^ mL/min (Figure 1C). The multiplication capacity, latent time, and burst size of phage vABWU2101 were determined by a one-step growth curve. The latent period of phage vABWU2101 was 20 min, and the burst size was approximately 283 phages/infected bacterial cell (Figure 1D). The lytic activity of phage vABWU2101 against the host strain was determined at different MOIs for 10 h. The results showed that the growth of uninfected MDR *A. baumannii* continuously increased (Figure 1E). A reduction in phage vABWU2101-infected MDR *A. baumannii* at MOIs of 0.01, 0.1, 1, and 10 was observed at 1 h post incubation. The growth of phage-infected MDR *A. baumannii* at MOIs of 0.1, 1, and 10 significantly decreased at 2 h post-incubation (*p* = 0.0007). A significant reduction in infected MDR *A. baumannii* at an MOI of 0.01 was detected at 3 h post incubation (*p* = 0.0058). The development of bacteria resistant to phage vABWU2101 was detected at 9 h post incubation.

### 3.4. Thermal, pH, UV Radiation and Long-Term Stability Studies

**Figure 2:**
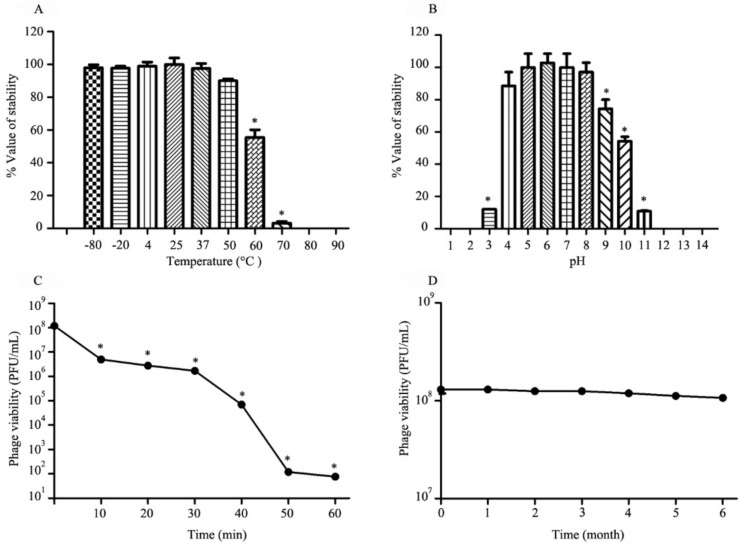
Stability of phage vABWU2101 under varied conditions. (**A**) Effect of temperature on the stability of phage vABWU2101. The phage was incubated at different temperatures for 2 h. (**B**) Effect of pH on the stability of phage vABWU2101. The phage was incubated for 2 h under different pH values. (**C**) Effect of UV on the stability of phage vABWU2101. (**D**) Effect of long-term storage at 4 °C on the stability of phage vABWU2101. Experiments were undertaken independently in duplicate with duplicate assay. The data show the mean ± SD (* *p* value < 0.05).

The stability of phage vABWU2101 under varied conditions was tested. The thermal stability of the phage was evaluated at −80 to 90 °C for 2 h. The results showed no significant reduction in phage stability after incubation at a temperature range of −80 to 50 °C (*p* = 0.0768) (Figure 2A). A significant reduction in phage activity was observed at 60 to 70 °C (*p* = 0.0094). There was no viable phage after incubation at 80 to 90 °C. For the sensitivity of phage to different pH values, the phage was exposed to varying pH values ranging from 1 to 14 for 2 h. There were no significant changes in phage viability at pH 4 to 8 (*p* = 0.4447) (Figure 2B). The stability of the phage was found to be significantly reduced at pH 3, 9, 10, and 11 (*p* = 0.0371), while the phage was completely inactivated at pH ≤ 2 and ≥12. In the case of phage stability under UV radiation, the viability of the phage was significantly reduced by approximately 1.62 log at 10 min post-UV exposure (*p* = 0.0012) (Figure 2C). After 60 min post-UV exposure, the viability of the phage was less than 10^2^ PFU/mL (0.00005%). For the stability of phage vABWU2101 under long-term storage at 4 °C, the phage viability was evaluated monthly. There was no significant reduction in the phage stability after 6 months (*p* = 0.1147) (Figure 2D).

### 3.5. Whole Genome Analysis and Annotation

**Figure 3:**
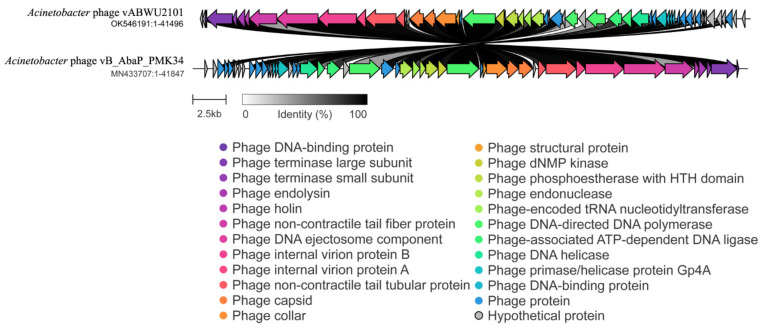
Map of the genome organization of phage vABWU2101. Visualization was performed by using Clinker. The coding sequences of phage vABWU2101 were compared with *Acinetobacter* phage vB_AbaP_PMK34, the most closely related phage. Homologous coding sequences are in the same color, and the homologous coding sequences between phage vABWU2101 and phage vB_AbaP_PMK34 are linked through gray bars with the percentage amino acid identity. The identity is represented by gray bars, with darker shades of gray indicating higher percentage identity.

The whole genome of phage vABWU2101 was characterized. The analysis revealed that the genome of phage vABWU2101 was 41,496 base pairs in size, with a GC content of 39.5%. The genome contained 57 predicted genes, encoding 57 predicted proteins (Figure 3). A total of 56 predicted genes started with a start codon ATG, while one predicted gene had a start codon TTG. No tRNA or antibiotic resistance genes were found in the genome. The functions of each gene were predicted by searching using BlastX against the NCBI database (Supplementary Table S1). The predicted genes were annotated as 26 proteins of known putative functional proteins and 29 hypothetical proteins. Two predicted genes were identified as hypothetical proteins of phage vABWU2101. The largest predicted gene was located in ORF9, which encoded internal virion protein C with 1032 amino acids, while the smallest predicted gene was located in ORF23, which encoded a hypothetical protein of phage vABWU2101 with 30 amino acids. The predicted proteins were categorized into three groups based on their functions. Nine predicted proteins were identified as phage-structure-related proteins, such as tailspike protein, internal virion protein C, internal virion protein B, putative internal virion protein A, tail tubular protein B, tail tubular protein A, putative capsid protein, head–tail connector protein, and structural protein. Fifteen predicted proteins involved in phage replication were DNA maturase B, putative DNA maturase, scaffolding protein, RNA polymerase, dNMP kinase, putative phosphoestherase, DNA endonuclease VII, tRNA nucleotidyltransferase, DNA exonuclease, DNA polymerase I, putative HNH homing endonuclease, ATP-dependent DNA ligase, DNA helicase, and DNA primase. The predicted proteins related to host lysis were endolysin and putative holin proteins. As several hypothetical proteins were predicted, the functions of predicted genes were further annotated by the RAST server. The predicted genes that were analyzed to encode hypothetical proteins by BlastX encoded 5 phage DNA-binding proteins, 7 phage proteins, and 17 hypothetical proteins (Supplementary Table S2). The DNA packaging mechanism of phage vABWU2101 was evaluated by the PhageTerm analysis [45]. Phage vABWU2101 packaged DNA by a headful (*pac*) mechanism (P1 type), and DNA was packaged in the reverse orientation.

**Figure 4:**
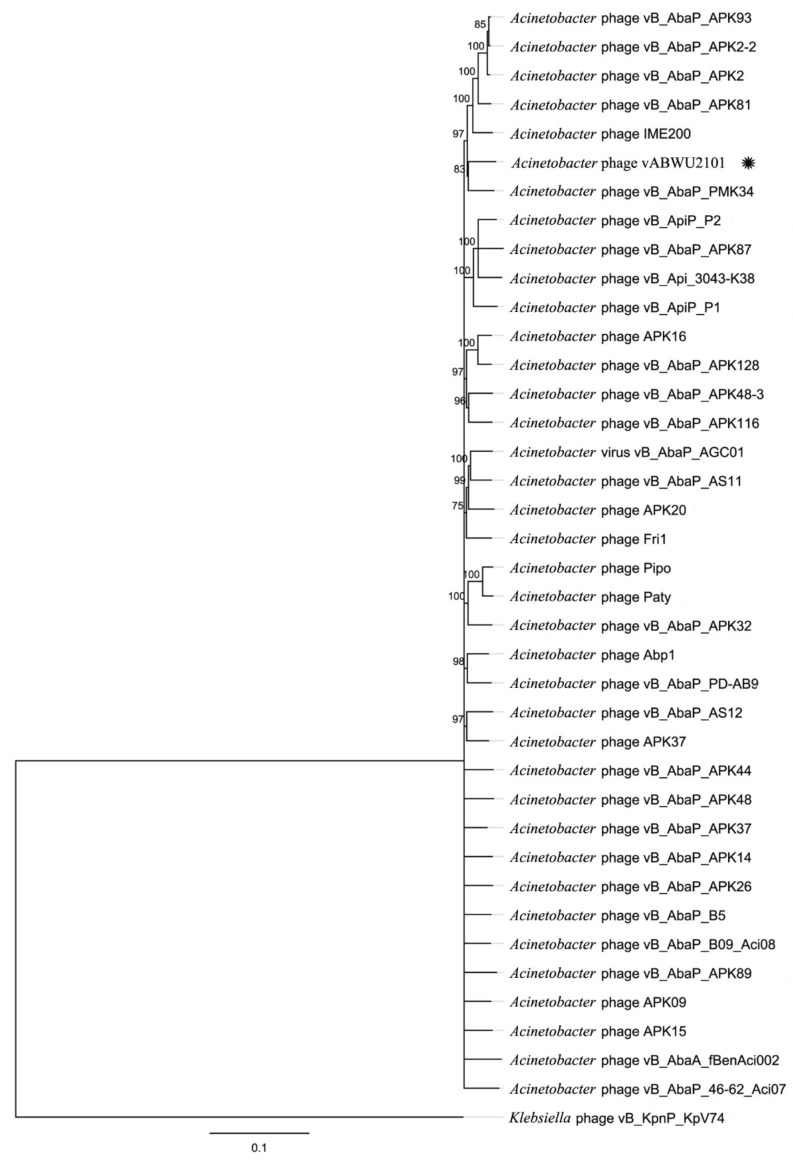
Phylogenetic tree analysis of phage vABWU2101 based on whole genome sequence comparisons of selected phages. The whole-genome phylogeny was constructed by the Geneious server using the neighbor-joining phylogenetic tree based on the JTT model with 500 bootstrap replications. Bootstrap percentages (>70) from the neighbor-joining method are represented at the nodes of the branches (above). The scale bar represents 10% nucleotide substitution percentage. Phage vABWU2101 is marked with an asterisk. *Klebsiella* phage vB_KpnP_KpV74 was used as an outgroup.

For comparative analysis of the phage genome, the genome of phage vABWU2101 was aligned by BlastN. The related phages were selected based on a certain sequence coverage range (>80%). The phylogenetic tree of phage vABWU2101 and the related phages was constructed (Figure 4). The genome of phage vABWU2101 shared the highest similarity at the DNA level with *Acinetobacter* phage vB_AbaP_PMK34 (MN433707.1), with 94.76% identity. *Klebsiella* phage vB_KpnP_KpV74 (NC_047811.1) was used as an out-group. The percent identity of phage vABWU2101 and phage vB_AbaP_PMK34 was less than 95% and the results suggested that phage vABWU2101 was considered a new species.

**Figure 5:**
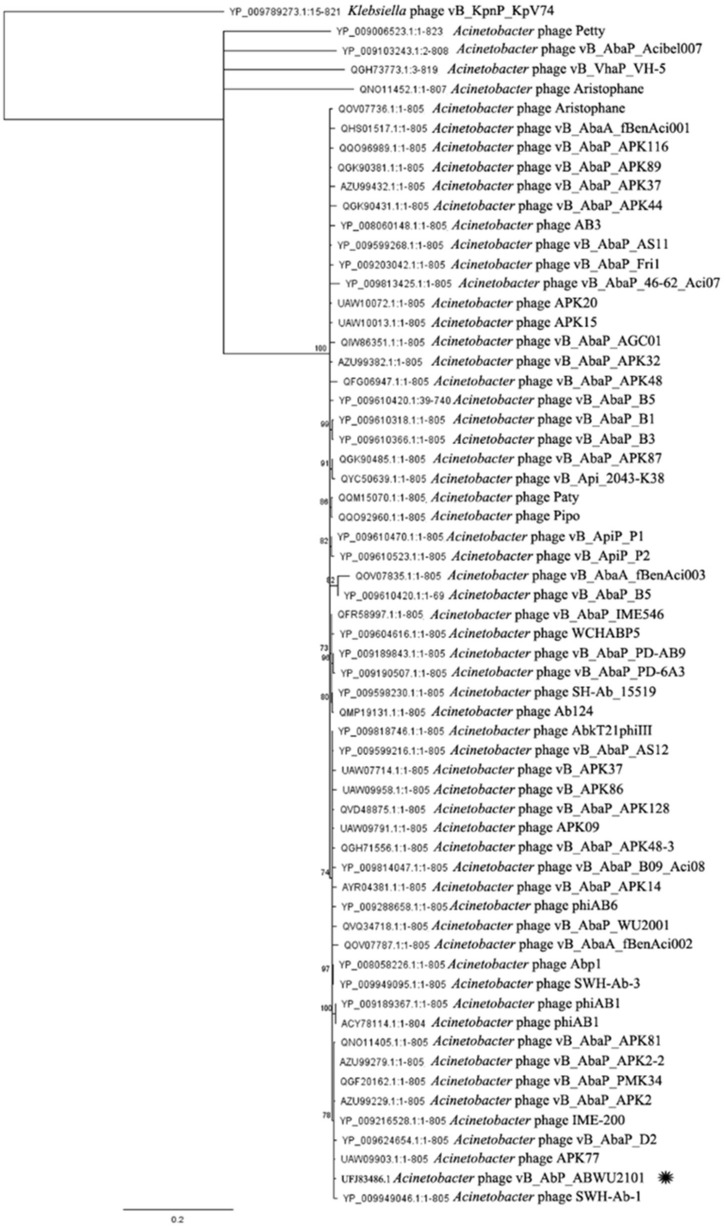
Comparative analysis of phage RNA polymerase proteins. The phylogenetic tree of RNA polymerase proteins was generated by the Geneious server using the neighbor-joining phylogenetic tree based on the JTT model with 1000 bootstrap replications. Bootstrap percentages (>70) from the neighbor-joining method are represented at the nodes of the branches (above). The scale bar represents 20% amino acid substitution. The query sequence is marked with an asterisk. RNA polymerase of *Klebsiella* phage vB_KpnP_KpV74 was used as an outgroup.

**Figure 6:**
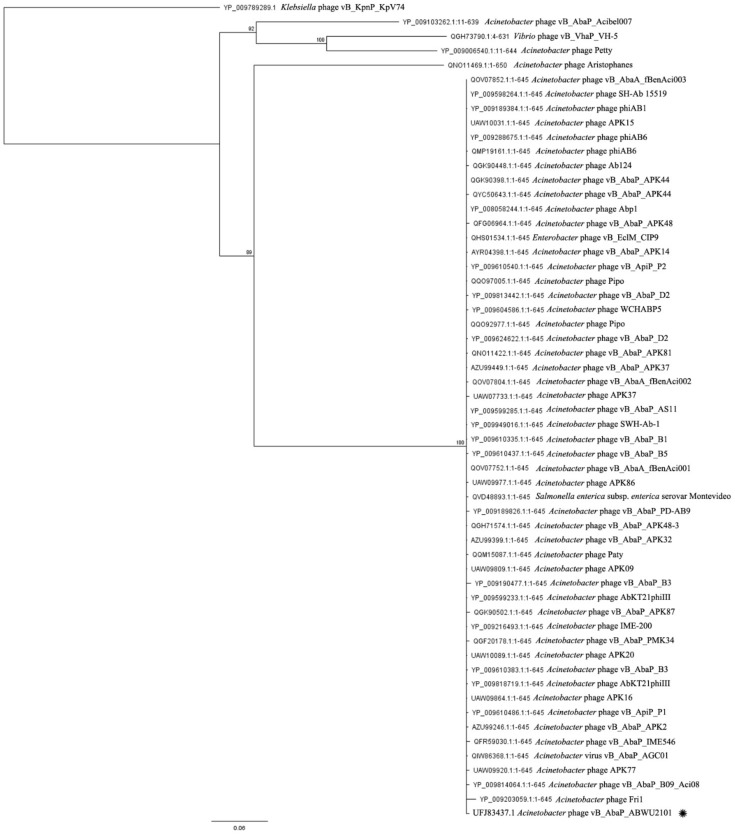
Comparative analysis of phage terminase large subunit proteins. The phylogenetic tree of phage terminase large subunit proteins was generated by the Geneious server using the neighbor-joining phylogenetic tree based on the JTT model with 1000 bootstrap replications. Bootstrap percentages (>70) from the neighbor-joining method are represented at the nodes of the branches (above). The scale bar represents 6% amino acid substitution. The query sequence is marked with an asterisk. Terminase large subunit of *Klebsiella* phage vB_KpnP_KpV74 was used as an outgroup.

To further understand the evolutionary relationships between phage vABWU2101 and other phages, RNA polymerase was selected for phylogenetic analysis with the Geneious server. The RNA polymerase of phage vABWU2101 was closely related to the RNA polymerase of *Acinetobacter* phage APK77 (UAW09903.1, 99.13% identity) (Figure 5). Phage terminase large subunit was also selected to investigate the genetic relationships of the phages. The phage terminase large subunit of phage vABWU2101 was closely related to putative DNA maturase B of *Acinetobacter* phage Fri1 (YP_009203059.1, 97.67% identity) (Figure 6). The results of genomic and phylogenetic tree analysis indicated that phage vABWU2101 was a new species in the *Friunavirus*, *Beijerinckvirinae*, family *Autographiviridae*, and order *Caudovirales*.

### 3.6. Anti-Biofilm Activity of Phage

**Figure 7:**
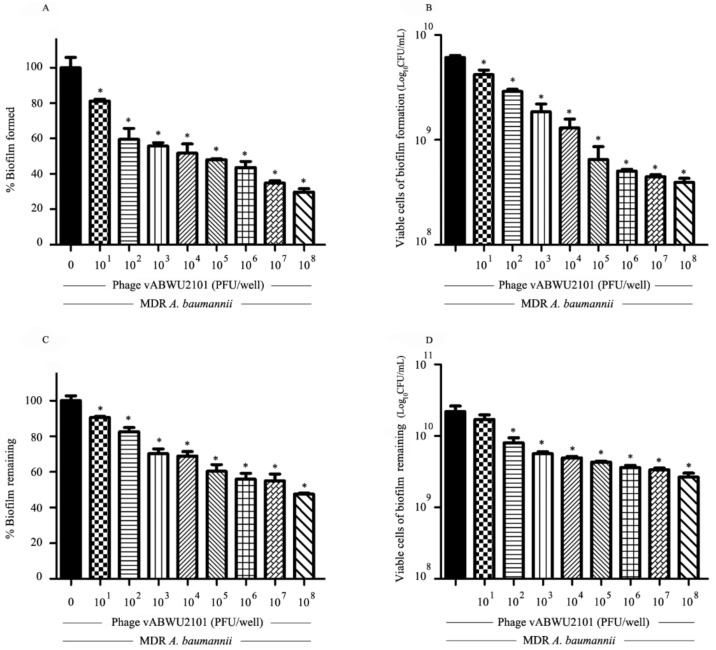
Antibiofilm activity of phage vABWU2101. Effect of phage vABWU2101 on the biomass of MDR *A. baumannii* biofilm formation (**A**) and MDR *A. baumannii* preformed biofilm (**C**). The effects of phage vABWU2101 at 10^1^–10^8^ PFU/well on biofilms were determined by crystal violet assay, and the dye was solubilized by ethanol. The absorbance was measured at 600 nm and presented as a percentage of biofilm compared to a control. Effect of phage vABWU2101 on bacterial cell viability of MDR *A. baumannii* biofilm formation (**B**) and MDR *A. baumannii* preformed biofilm (**D**). The viable cells in the biofilms were determined by the colony counting method. Experiments were undertaken independently in triplicate with duplicate assay. The data show the mean ± SD (* *p* value < 0.05).

The efficacy of phage vABWU2101 in antibiofilm formation and biofilm elimination was evaluated by standard crystal violet staining and cell viability counting. For assessing antibiofilm formation activity of the phage, MDR *A. baumannii* was cocultured with phage vABWU2101 in the wells, followed by incubation for 48 h. Phage vABWU2101 at 10^1^–10^8^ PFU/well significantly reduced biofilm biomass from 18.77 to 70.25% (*p* = 0.047) (Figure 7A). The viable bacterial cells were significantly decreased from 0.19 to 1.215 log compared to the negative treatment control (*p* = 0.0342) (Figure 7B). The ability of phage vABWU2101 to remove biofilms was also determined. MDR *A. baumannii* biofilm was allowed to form in the wells and then supplemented with the phage at different concentrations. Approximately 9.43 to 52.43% biomass reduction in preformed biofilm was observed after incubation with phage vABWU2101 at 10^1^ to 10^8^ PFU/well (*p* = 0.0408) (Figure 7C). Phage vABWU2101 at 10^2^ to 10^8^ PFU/well significantly removed 0.05 to 0.955 log of viable bacterial cells compared to the negative treatment control (*p* < 0.0474) (Figure 7D). The results indicated that phage vABWU2101 had antibiofilm properties against MDR *A. baumannii* in a dose-dependent manner.

### 3.7. Synergistic Activity of Phage vABWU2101 and an Antibiotic

The activity of tigecycline against MDR *A. baumannii* was investigated by MIC and MBC tests. The MIC and MBC of tigecycline were 2 and 8 μg/mL, respectively. Subsequently, the synergistic activity of phage vABWU2101 and tigecycline was evaluated. The MIC and MBC values of the combinations of tigecycline and phage vABWU2101 at an MOI of 0.1 or 1 were 0.065 μg/mL (1/32× MIC) and 1 μg/mL (1/2× MIC), respectively. The FIC index was calculated to assess synergism, additivism/indifference, or antagonism. The FIC index value of the phage-tigecycline combinations was 0.033, and the results were then interpreted based on the FIC index. The results suggested that the combination of phage vABWU2101 and tigecycline was synergistic.

### 3.8. In Vitro Activity of a Combination of Phage vABWU2101 and Tigecycline

**Figure 8:**
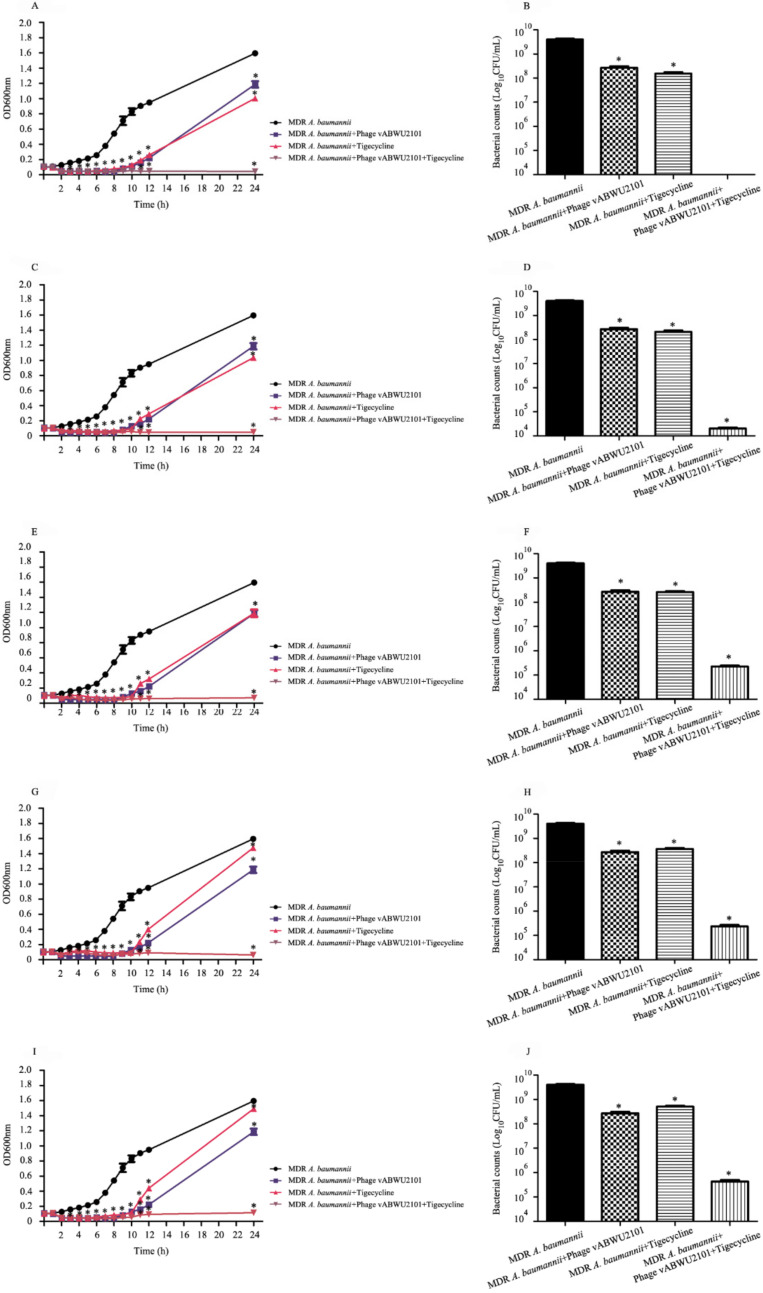
Synergistic antibacterial activity of phage vABWU2101 and tigecycline against MDR *A. baumannii*. Effect of the combination of phage vABWU2101 and tigecycline at 1/2× MIC (**A**,**B**), 1/4× MIC (**C**,**D**), 1/8× MIC (**E**,**F**), 1/16× MIC (**G**,**H**), and 1/32× MIC (**I**,**J**) on the MDR *A. baumannii* growth curve and bacterial cell viability, respectively. The efficacy of a combination of phage vABWU2101 and tigecycline against MDR *A. baumannii* was evaluated by OD600 measurement every hour for 12 h. At 24 h post incubation, the growth of MDR *A. baumannii* was determined using the colony counting method. Experiments were undertaken independently in triplicate with duplicate assay. The data show the mean ± SD (* *p* value < 0.05).

The efficacy of a combination of phage vABWU2101 and tigecycline against MDR *A. baumannii* was evaluated by OD600 measurement every hour for 12 h. At 24 h post incubation, the growth of MDR *A. baumannii* was detected, and bacterial cell viability was counted. The growth of untreated MDR *A. baumannii*, a control, continuously increased (Figure 8A,C,E,G,I). A significant reduction in phage vABWU2101- or tigecycline-treated MDR *A. baumannii* was observed at 2 h post incubation (*p* = 0.0026 and *p* = 0.0036, respectively). At 9 h post incubation, the growth of MDR *A. baumannii* incubated with only phage vABWU2101 or only tigecycline was increased. However, the growth of MDR *A. baumannii* after incubation with only phage vABWU2101 or only tigecycline was significantly lower than that of the untreated control at 24 h post incubation (*p* = 0.0103 and *p* = 0.0043, respectively). For the combination of the phage and tigecycline, the growth of MDR *A. baumannii* significantly declined after incubation with the combination of phage vABWU2101 and tigecycline at 1/2–1/32× MIC for 2 h compared to the untreated control (*p* = 0.0024) (Figure 8A,C,E,G,I). At 24 h post-incubation, the growth of MDR *A. baumannii* after incubation with the combination of phage vABWU2101 and tigecycline was significantly lower than that of the untreated control, only phage vABWU2101- or only tigecycline-treated MDR *A. baumannii*. The bacterial cell viability was also determined. The viability cell count of phage vABWU2101-infected MDR *A. baumannii* at an MOI of one was decreased by 1.13 log (p = 0.0029) (Figure 8B,D,F,H,J). The reduction in bacterial cell viability of tigecycline-treated MDR *A. baumannii* at 1/2–1/32× MIC was lower than that in the untreated control by 1.245 to 0.89 log (*p* = 0.0033). Significant reductions of approximately 5.195 to 3.965 log in MDR *A. baumannii* cell viability were observed after incubation with the combination of phage vWUAB2101 and tigecycline at 1/4–1/32× MIC, compared to MDR *A. baumannii*, phage, or tigecycline alone (*p* = 0.0025) (Figure 8B,D,F,H,J). There were no viable bacteria after incubation with the combination of phage vWUAB2101 and tigecycline at 1/2× MIC (Figure 8B). The results confirmed that the combination of phage vABWU2101 and tigecycline was synergistic.

### 3.9. Activity of the Combination of Phage and Tigecycline against Biofilms

**Figure 9:**
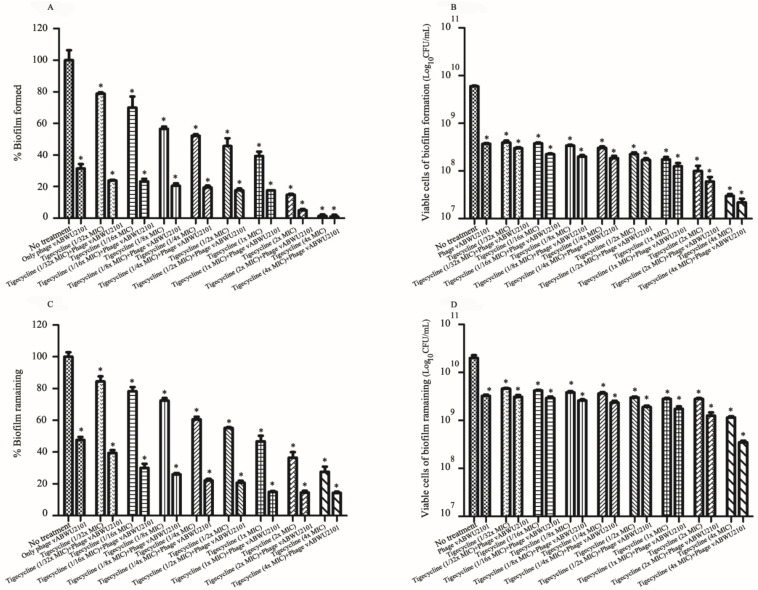
Antibiofilm activity of synergistic combinations of phage vABWU2101 and tigecycline. Biomass (**A**) and bacterial cell viability (**B**) of MDR *A. baumannii* biofilm formation treated with a combination of phage vABWU2101 at an MOI of 1 and tigecycline at 1/32–4× MIC. Biomass (**C**) and bacterial cell viability (**D**) of MDR *A. baumannii* preformed biofilms treated with a combination of phage vABWU2101 at an MOI of 1 and tigecycline at 1/32–4× MIC. The effects of a combination of phage vABWU2101 and tigecycline on biofilms were determined by crystal violet assay. The effect of a combination of phage vABWU2101 and tigecycline on the bacterial cell viability of MDR *A. baumannii* biofilms were determined by the colony counting method. Experiments were undertaken independently in triplicate with duplicate assay. The data show the mean ± SD (* *p* value < 0.05).

The antibiofilm activity of synergistic combinations of phage vABWU2101 and tigecycline was evaluated by standard crystal violet staining and cell viability counting. For assessing antibiofilm formation activity of the combination of phage and tigecycline, phage vABWU2101 at an MOI of 1 reduced biofilm’s biomass by 68.55% and bacterial cell viability by 1.225 log compared to a control, MDR *A. baumannii* without treatment (*p* = 0.005 and *p* = 0.0007, respectively) (Figure 9A,B). Tigecycline treatment alone at 1/32× MIC to 4× MIC decreased biofilm biomass by 21.05–98.55% (*p* = 0.0429) and bacterial cell viability by 1.2 to 2.295 log (*p* = 0.0007). The biomass of MDR *A. baumannii* biofilm formation was significantly reduced by 76.18–98.95% after treatment with the combination of phage vABWU2101 and tigecycline at 1/32× MIC to 4× MIC compared with the control (*p* = 0.0035) (Figure 9A). The bacterial cell viability after the combination treatment was significantly reduced by 1.295 to 2.395 log *(p* = 0.0007) (Figure 9B). For biofilm removal activity of the combination of phage and tigecycline, phage vABWU2101 at an MOI of 1 removed 52.39% of the biofilm biomass *(p* = 0.0022) and 0.875 log of the bacterial cell viability (*p* = 0.014) (Figure 9C,D). The tigecycline treatment alone reduced the biofilm biomass by approximately 15.58–72.36% (*p* = 0.0357) and the bacterial cells by 0.74 to 1.085 log *(p* = 0.0165). The combination of phage vABWU2101 plus tigecycline at 1/32× MIC to 4× MIC removed 60.55–85.68% of the biofilm biomass compared to phage or tigecycline treatment alone *(p* = 0.0015) (Figure 9C). A significant reduction in bacterial cell viability was observed by approximately 0.89 to 1.85 log *(p* = 0.0139) (Figure 9D). The results indicated that the combination of phage and tigecycline exhibited synergistic antibiofilm activities.

### 3.10. Scanning Electron Microscopy Analysis of Bacterial Surface Structures

**Figure 10:**
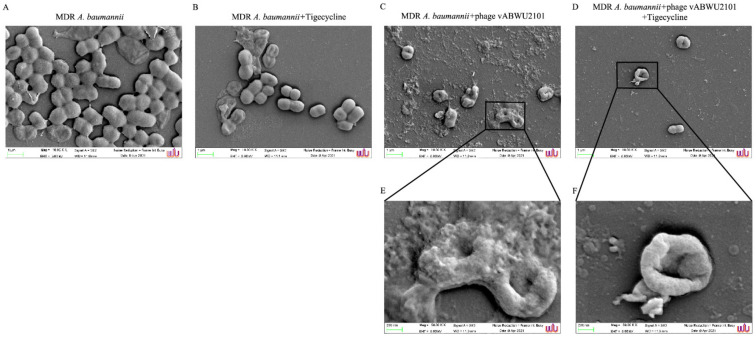
Ultrastructural analysis of *A. baumannii* treatment observed by FE-SEM at magnifications of ×10,000 and ×50,000. (**A**) MDR *A. baumannii.* (**B**) MDR *A. baumannii* treated with tigecycline at 1/32× MIC. (**C**,**E**) MDR *A. baumannii* treated with phage vABWU2101 at an MOI of 1. (**D**,**F**) MDR *A. baumannii* treated a combination of phage vABWU2101 at an MOI of 1 and tigecycline at 1/32× MIC.

The effects of phage vABWU2101 combined with tigecycline on MDR *A. baumannii* cells were observed under SEM. The untreated MDR *A. baumannii* cells displayed rod-shapes with smooth surfaces (Figure 10A). A slight change in the cell membrane of MDR *A. baumannii* treated with only tigecycline at 1/32× MIC was observed, and the number of bacterial cells was slightly reduced compared with untreated samples (Figure 10B). SEM micrographs of MDR *A. baumannii* treated with only phage vABWU2101 at an MOI of 1 (Figure 10C,E) or the combination of phage vABWU2101 at an MOI of 1 and tigecycline at 1/32× MIC (Figure 10D,F) showed morphological changes and disruption of the cell membrane, such as membrane shrinkage and pitting, leading to cell death. Lower numbers of bacterial cells were detected in MDR *A. baumannii* treated with the combination compared to cells that were untreated or treated with only phage or only tigecycline.

## 4. Discussion

*A. baumannii* is an opportunistic pathogen with increasing relevance in community-acquired and hospital-acquired infections. *A. baumannii* has been implicated in various infections, including infections of the skin and soft tissues, respiratory infection, meningitis, urinary tract, and sepsis. Currently, *A. baumannii* has emerged as a significant MDR nosocomial pathogen worldwide. The rapid emergence of antimicrobial resistance in *A. baumannii* has been reported, and thus *A. baumannii* is one of the bacteria in the critical list of superbugs that has been published by the World Health Organization [56]. Therefore, the development of new approaches and strategies to control MDR *A. baumannii* has been of interest to investigate.

In the present study, a novel phage specific for MDR A. baumannii-associated SSTIs was isolated and characterized, followed by evaluation of antimicrobial activity in vitro. Phage vABWU2101 was isolated from environmental water, and the phage was a member of the *Podoviridae* or *Autographiviridae* family and the order *Caudovirales* based on the TEM morphology of the phage particles. However, the phage classification was confirmed by genomic analysis. Several *Podoviridae* or *Autographiviridae* phages specific for *A. baumannii* have been studied, such as phage SH-Ab 15497 [57], phage AbTJ [58], phage vB_AbaP_AS11, phage vB_AbaP_AS12 [59], and phage Aristophane [60]. Based on the spot test and the EOP analysis, phage vABWU2101 was found to be specific for *A. baumannii* strains and had a narrow host range activity. Each phage has a different growth profile that can be used to categorize the lytic phage and evaluate the infectivity of the therapeutic phage [61]. Phage vABWU2101 could adsorb into host cells rapidly. Previous studies on *Podoviridae* and *Autographiviridae phages* showed a latency period of 15–90 min and burst size of 70–300 particles per infected cell [58,59]. Phage vABWU2101 had a short latency period and a large burst size when compared with these earlier reports. These characteristics make the phage suitable for biocontrol strategies. Environmental factors are important factors affecting phage stability and the effectiveness of treatment in phage therapy [62]. The integrity of the phage should be maintained in an appropriate range of temperatures and pH values to stabilize its viability. The sensitivity of phages has been evaluated in many environmental conditions. Phage vABWU2101 showed good stability under a wide range of temperatures and pH values. UV radiation is an important condition that can destabilize phage activity in the natural environment and the application of phage therapy [63]. Phage vABWU2101 was sensitive to UV radiation similar to many phages in earlier studies, such as phage-BF25/12-specific *Dickeya* species [64] and phage-KP1801-specific *K. pneumoniae* [34]. However, phage vABWU2101 still had activity after exposure to UV radiation for 60 min. When the phages are developed as phage products, the phages are preserved and can undergo a loss of stability during long-term storage [65]. Phage vABWU2101 showed a high stability after storage at 4 °C for 6 months. The ability of phages to survive under broad environmental conditions suggested that it was possible to apply phage vABWU2101 as a biocontrol agent.

Whole-genome sequencing was performed to determine the genetic profile of the phage. The whole-genome sequencing results revealed that phage vABWU2101 was a novel phage in the Autographiviridae family, order *Caudovirales.* In previous reports, *Autographiviridae* phages were reported to have genome sizes in the range of 39,425 to 47,868 base pairs [60,66]. The genome size of phage vABWU2101 was in the range of these earlier reports. The predicted genes were annotated by BlastX and RAST servers. Among 57 predicted genes, a large portion of the genome (29.82%) represented hypothetical proteins, while 70.18% shared sequence identities to the genes with known functions. The gene annotation showed that the predicted proteins with known functions were grouped into four clusters: 9 proteins with phage structural functions, 20 proteins with DNA replication/modification functions, 2 proteins with bacterial infection functions, and 5 phage proteins. There were no toxin-related genes, and antimicrobial resistance genes. Moreover, there was no virulence gene or prophage-related genes such as integrase and repressor in the phage genome according to PHASTER and Prophage Hunter online search tools [67,68]. The genetic relationships of phage vABWU2101 and related phages were investigated by phylogenetic tree analysis, and the results indicated that phage vABWU2101 was a novel lytic phage in the genus *Friunavirus*, subfamily *Beijerinckvirinae*, family *Autographiviridae*, and order *Caudovirales*. These results indicate the potential suitability of this phage for use in phage therapy. Most lytic phages encode specific proteins that play a role in replication. The evolutionary relationship among phages was evaluated by phylogenetic tree analysis of specific genes. The amino acid sequences of the specific genes were aligned by multiple sequence alignment, and phylogenetic trees were constructed. RNA polymerase, an enzyme associated with transcription initiation and elongation factors, was selected as a model transcription module [69]. Phage vABWU2101 was shown to contain only one gene-encoding RNA polymerase, and the gene shared the highest percentage of amino acid similarity with *Acinetobacter* phage APK77. The results indicated that phage vABWU2101 had the same transcription strategy as phage APK77. In addition, the phage terminase large subunit was used as a model for the phylogenetic tree of DNA packaging-related gene. The phage terminase large subunit shared high amino acid sequence similarity with the DNA maturase B of *Acinetobacter* phage Fri1. The results obtained confirm that phage vABWU2101 might use the same DNA packaging mechanism as phage Fri1. Due to the size of the phage genome, phage vABWU2101 had a small genome size compared to the previous reports on *Acinetobacter* phages [70,71,72]. Many ORFs on the genome of phages are usually encoding hypothetical proteins, and some of them could not be aligned to any genes in the available databases. The unknown genes may encode unwanted genes such as the unknown toxins and antibiotic-resistant genes. Thus, the smaller phage genome is better for therapy, indicating that phage vABWU2101 might be suitable for applications.

To initiate colonization and infection, *A. baumannii* can colonize and form biofilms on abiotic surfaces such as materials (polystyrenes and glass), and biotic surfaces, such as epithelial cells and fungal surfaces [73]. Biofilms, a bacterial community covered with extracellular polymeric substances (EPS), can evade host immune defense and persist during antibiotic treatment. Thus, biofilm infections are difficult to handle, and biofilms show much greater resistance to antibiotics than planktonic bacterial cells [74]. Several phages have been employed to combat biofilms caused by *A. baumannii*. For example, phage ISTD, specific to carbapenem-resistant *A. baumannii*, has been shown to decrease the number of viable bacteria within biofilms [75]. Phage AB7-IBB1 infection of *A. baumannii* inhibited biofilm formation on abiotic and biotic surfaces [76]. Based on our plaque morphology result, the plaque of phage vABWU2101 exhibited a halo surrounding the plaque, indicating depolymerase activity of the phage [75]. Phages with depolymerase activity can depolymerize polysaccharides such as EPS, lipopolysaccharide (LPS), and capsular polysaccharide (CPS), key biofilm components for biofilm formation, following attachment of the phage to the bacterial host receptor and subsequent infections. In addition to its function as a phage receptor-binding domain, another function of the tail spike protein has been reported to be associated with its depolymerase activity [77,78]. Previous studies showed that CPS depolymerase, derived from the tail spike protein of phage phiAB6, could reduce *A. baumannii* biofilms [79]. Our whole-genome analysis revealed that phage vABWU2101 encoded a tail spike protein, indicating the possibility of evaluating antibiofilm activity. To clarify the depolymerase domain in the phage genome, the functional analysis of phage vABWU2101-encoded tail spike protein was analyzed by the InterPro web server. A pectase lyase domain, the domain associated with phage depolymerase activity, was detected, indicating that the phage vABWU2101 gene encodes a protein with depolymerase activity [80]. Subsequently, the antibiofilm efficacy of the phage was evaluated. Our study showed that phage vABWU2101 could combat both preformed biofilm and biofilm formation and the phage could also significantly reduce bacterial viability. Using phage, or phage-encoded enzymes might be useful for controlling biofilms and developing biomedical applications [81].

During the phage infection process, bacterial cells can develop a resistance mechanism for blocking phage adsorption to cell receptors and infection [82]. Several studies on the development of bacterial resistance to phages during phage therapy have been reported [83]. In this study, we found that the growth of phage vABWU2101-infected MDR *A. baumannii* cells was increased after 9 h, indicating the emergence of phage-resistant bacteria. To completely remove bacteria and enhance the efficiency of the phage or antibiotic, the synergistic interactions of phage vABWU2101 and antibiotic were investigated. The host of phage vABWU2101 was isolated from skin infections, and the host stain was sensitive to tigecycline. Thus, tigecycline, the approved antibiotic for the treatment of skin infections, was selected as the model antibiotic in this study [14]. Tigecycline antibiotic is a potent bacteriostatic agent in the treatment of Gram-positive and Gram-negative bacteria [21]. The phage vABWU2101 and tigecycline combination exhibited synergism. Another experiment with time-kill and biofilm assays was performed to evaluate the antibacterial and antibiofilm activities of this combination. Our results showed that phage vABWU2101 demonstrated synergistic activity with tigecycline to combat planktonic MDR *A. baumannii* and biofilm cells. Phage-tigecycline synergy was also evaluated by electron microscopy. The results of this study confirmed that tigecycline could be combined with phage vABWU2101 to improve the efficacy of MDR *A. baumannii* killing. This combined approach will shed light on overcoming the bacterial resistance development during treatment. Supplementing tigecycline with phage vABWU2101 might enable the development of a new therapeutic product for SSTIs and diabetic foot ulcer infections.

In conclusion, a novel phage, vABWU2101, isolated in this study exhibited efficient bactericidal and antibiofilm activities toward MDR *A. baumannii* in vitro. The phage and antibiotic combination showed promising synergistic effects.

## Figures and Tables

**Table 1:** Host range infection and EOP of phage vABWU2101.

Strain	Phage vABWU2101	
	Lytic Activity	EOP
MDR *A.* *baumannii* ABPW0181	+	High (0.64)
MDR *A.* *baumannii* ABPW0182	+	High (0.55)
MDR *A.* *baumannii* ABPW0183	-	-
MDR *A.* *baumannii* ABPW0184	-	-
MDR *A.* *baumannii* ABPW0185	+	High (Host = 1)
MDR *A.* *baumannii* ABPW0186	+	Moderate (0.17)
MDR *A.* *baumannii* ABPW0187	+	Moderate (0.21)
MDR *A.* *baumannii* ABPW0188	-	-
MDR *A.* *baumannii* ABPW0189	+	Low (0.08)
MDR *A.* *baumannii* ABPW0190	+	High (0.91)
MDR *A.* *baumannii* ABPW0191	+	Low (0.09)
MDR *A.* *baumannii* ABPW0192	+	Moderate (0.32)
MDR *A.* *baumannii* ABPW0193	+	Moderate (0.48)
MDR *A.* *baumannii* ABPW0194	-	-
MDR *A.* *baumannii* ABPW0195	+	High (0.72)
MDR *A.* *baumannii* ABPW0196	+	Moderate (0.27)
MDR *A.* *baumannii* ABPW0197	+	High (0.93)
MDR *A.* *baumannii* ABPW0198	-	-
MDR *A.* *baumannii* ABPW0199	-	-
MDR *A.* *baumannii* ABPW0200	+	Low (0.06)
*K. pneumoniae*	-	-
MRSA	-	-

+ able to produce lytic zone, - was unable to produce lytic zone. The EOP values were classified into 4 groups: high production (a ratio ≥ 0.5), moderate production (a ratio 0.1 ≤ EOP < 0.5), low production (a ratio 0.001 < EOP < 0.1), and no production (a ratio ≤ 0.001). Experiments were undertaken independently in duplicate with duplicate assay.

## Data Availability

All datasets presented in this study are included in the article.

## Supplementary Materials

The following are available online at https://www.mdpi.com/article/10.3390/v14020194/s1: Table S1: Annotation of the specific function of ORFs in phage vABWU2101 genome was conducted using BlastX; Table S2: Annotation of the specific function of ORFs in phage vABWU2101 genome was conducted using the RAST database.
